# Acoustic Analysis of PD Speech

**DOI:** 10.4061/2011/435232

**Published:** 2011-10-03

**Authors:** Karen Chenausky, Joel MacAuslan, Richard Goldhor

**Affiliations:** Speech Technology and Applied Research Corporation, Bedford, MA 01730-1417, USA

## Abstract

According to the U.S. National Institutes of Health, approximately 500,000 Americans have Parkinson's disease (PD), with roughly another 50,000 receiving new diagnoses each year. 70%–90% of these people also have the hypokinetic dysarthria associated with PD. Deep brain stimulation (DBS) substantially relieves motor symptoms in advanced-stage patients for whom medication produces disabling dyskinesias. This study investigated speech changes as a result of DBS settings chosen to maximize motor performance. The speech of 10 PD patients and 12 normal controls was analyzed for syllable rate and variability, syllable length patterning, vowel fraction, voice-onset time variability, and spirantization. These were normalized by the controls' standard deviation to represent distance from normal and combined into a composite measure. Results show that DBS settings relieving motor symptoms can improve speech, making it up to three standard deviations closer to normal. However, the clinically motivated settings evaluated here show greater capacity to impair, rather than improve, speech. A feedback device developed from these findings could be useful to clinicians adjusting DBS parameters, as a means for ensuring they do not unwittingly choose DBS settings which impair patients' communication.

## 1. Introduction

### 1.1. Background

Parkinson's disease (PD) is an idiopathic neurodegenerative disease caused by loss of dopamine-producing cells in the substantia nigra of the basal ganglia, affecting over one-half million people in the U.S., most over age 50. Its major symptoms are muscular rigidity, bradykinesia, resting tremor, and postural instability. An estimated 70%–90% of patients with PD also develop speech or voice disorders [1] specifically hypokinetic dysarthria [2, page 174]. Hypokinetic dysarthria is characterized by monopitch, monoloudness, underarticulation, and harsh and/or breathy voice. It worsens with disease severity and duration [3] so that patients who are more incapacitated and more reliant on caregivers are also more difficult to understand.

The major treatment for Parkinson's disease is L-dopa, a dopamine precursor given orally to patients. L-dopa is most effective on the general motor symptoms of PD, with variable effects on speech. For example, some studies [4] have found that though motor performance, vocal tremor, and glottal vibration were improved in patients with PD after taking L-dopa, there were no significant improvements in prosody, articulation, or vocal intensity after medical therapy. Other researchers [5] have assessed the amount of pause time in speech, the speech rate, the articulation rate, and the standard deviation of the fundamental frequency of patients with PD and compared them to age-matched normal controls. After medication with L-dopa, only the pause time of the patients with PD had improved though it was within the normal range both on and off medications. Still others [6, 7] found significant improvements in pitch and loudness variation in patients with PD after medication as well as an improvement in intelligibility. Respiratory parameters such as vital capacity, length of sustained vowel phonation, and phonated quotient were also improved. There was no correlation between improvement in respiratory parameters and intelligibility, but there was an increase in loudness and a faster speech rate when patients with PD were on medication, though the intensity of their speech decayed more quickly [8]. The authors conclude that the changes in speech caused by L-dopa therapy may or may not be advantageous to individual patients, depending on their specific symptom profile.

Further, medical therapy can only be used so long. Once the number of substantia nigra cells decreases past a critical point, medication causes large and uncomfortable fluctuations in motor function in patients with PD. These fluctuations consist of levodopa-induced involuntary movements (dyskinesias) followed by a return of rigidity. Electrically stimulating the subthalamic nucleus (STN) has been shown to reduce the rigidity, bradykinesia, and tremor of PD by inhibiting the activity of the STN in the basal ganglia-thalamus loop. Within the past decade, deep brain stimulation (DBS) of the subthalamic nucleus (STN) has provided substantial clinical benefit to patients with PD whose disease has become difficult to manage by medication alone [9]. Patients whose major symptom is tremor generally have a better prognosis for DBS than do those who suffer more from rigidity or akinesia. Patients who develop psychiatric symptoms of PD such as hallucinations or cognitive disturbances are considered less appropriate candidates for DBS therapy. Side effects of the DBS implantation surgery itself, such as hematoma or paralysis, are rare [10]. Instances of hardware problems such as lead fracture, dislocation of the leads, and insertion site infection are more common [10].

As with L-dopa therapy, most of the focus of DBS treatment has been directed toward the motoric features of PD. For example, it has been suggested that the three main goals of DBS programming should be to maximize relief from symptoms, to minimize side effects, and to preserve battery life (in that order) [11]. Much less emphasis has been placed on other components of PD, such as postural instability or speech. Yet, hypokinetic dysarthria eventually appears in the majority of the PD population and frequently compromises a patient's quality of life by impairing a person's ability to communicate effectively with family members and health care providers [12, page 438; 32]. While DBS has been shown to reduce tremor, bradykinesia, and gait problems in patients with PD, its specific effects on speech are equivocal. Some studies report that voice function improves in parallel with motor symptoms after DBS treatment, while others report a worsening in speech in the setting of motor symptom improvement following DBS surgery. Still, others report no net effect on speech. Recently, a more nuanced view of the effects of DBS on speech has emerged. 

Studies reporting a positive effect on speech of DBS therapy start with [13]. Using UPDRS item 18, which asks the practitioner rating the patient to score speech as “0-normal, 1-slight loss of expression, diction, or volume, 2-monotone, slurred but understandable, moderately impaired, 3-marked impairment, difficult to understand, or 4-unintelligible”, these researchers rated patients with PD on and off medication, prior to surgery, then again after surgery. Off medication, the patients' scores averaged 2.8 (in the moderately-to-markedly impaired range), but medicated, scores averaged 1.2 (closer to minimally impaired). Once the DBS implants had been “optimally programmed”, patients averaged 1.5 on stimulation/off medications. With both medication and stimulation, scores averaged 1.1. In this study, therefore, there was a mild-to-moderate improvement in speech, according to doctors' perceptions, on stimulation and medication. Likewise, [14] found a “tendency for voice to improve” in patients on stimulation. More specifically, patients' repetitions of a nonsense word were shorter when they were receiving stimulation, and in running speech, their pause duration was shorter. Their maximum phonation time was also increased on stimulation. Finally, [15] confirmed previous findings of longer maximum phonation time and lower pause time on stimulation than off. In addition, they found less jitter (cycle-to-cycle amplitude variation) and shimmer (cycle-to-cycle frequency variation), and better relative stability of F0.

Negative effects of DBS on speech were found by [1], where speech pathologists (SLPs) rated 32 dimensions of speech and voice performance on readings of the “Grandfather Passage” on and off stimulation. Ratings on 22 of the dimensions moved away from the control means on stimulation as compared to off stimulation. And [16] used two questions from the UPDRS, 5 and 18 (patient and practitioner ratings of speech performance, resp.), to assess changes in speech after stimulation. Ratings of speech were approximately one point worse on stimulation than off though (in agreement with [14, 15]) maximum phonation time was longer by approximately 1.5 seconds. Ratings of “normal” or “abnormal” on patients' conversational speech also worsened on stimulation. 

Other teams of researchers examined longer-term effects of DBS on speech. The researchers in [17] used UPDRS item 18 ratings at one, three, and five years after implantation. Not only was speech the only function whose score off medication failed to improve over time, but the Year 5 speech score on both medication and stimulation was significantly worse than the Year 1 speech score. Clinicians in [18] found similar deterioration in UPDRS item 18 scores over time. Though average scores for their patients one year after surgery were 1.0 off stimulation and medication versus 0.9 on stimulation (a nonsignificant improvement), after four years, the on-stimulation and off-stimulation scores were the same at 1.6. On-stimulation scores from Year 4 were significantly worse than their Year 1 counterparts, as were off-stimulation Year 4 scores compared to Year 1 off-stimulation scores.

But many other studies have found no net effect of DBS therapy on speech. Some, using UPDRS items 5 and 18 and a dysarthria test, found that only two of seven patients performed significantly worse on stimulation and medication than presurgery on medication [19]. No significant changes were noted for the other five patients. Others found no significant changes on the CAIDS (Computerized Assessment of Intelligibility of Dysarthric Speech) on patients three months after surgery and on DBS therapy, compared to their performance one month before surgery [1]. Using the same test, [20] found no change in mean CAIDS score after one year for DBS or medication-only groups of patients, though vocal intensity did increase after one year for patients receiving both medical and surgical therapies (as compared to presurgery on-medication). Still others also found two groups of patients, one of which showed improved speech and the other of which showed worsened speech as a result of DBS therapy [20]. In this study, there was an overall decrease of approximately 15% on CAIDS scores off-medication and on-stimulation. 

To understand why there might be positive, negative, or no effects on speech of DBS, many research teams are using more critical analyses: looking at individual patients, laterality of stimulation, or different settings. For example, [4] found that in their patients, UPDRS item 18 was always rated 2 (moderate impairment) in all four combinations of medical and DBS therapy. Looking at 49 patients across multiple institutions, [10] reported only nine patients with speech difficulties as a result of DBS implantation. These changes correlated best with disease duration and the presence of axial symptoms (such as balance disturbances). 

Because the presence of deteriorated or improved speech seems to depend heavily on the individual patient's constellation of symptoms, [21] specifically studied four patients of different ages and disease durations. Again using UPDRS item 18, they found that one patient showed significant dysarthria off medications and stimulation, but was able to produce a short phonation when receiving either therapy. A second patient experienced dysphonia with no therapy, but on stimulation or medication experienced a worsening in dysarthria. Receiving both therapies together resulted in even worse dysarthria. By contrast, a third patient's speech was less dysarthric on stimulation but worse with too much of either therapy. The fourth patient's speech was worse with medical and DBS therapy, especially when the stimulation was more caudal, suggesting more corticobulbar involvement. The author drew two conclusions: First, “Item 18 of the UPDRS does not adequately evaluate the often complex speech changes that may result from L-dopa therapy or STN stimulation.” (page 1513). Second, he noted that many patients are forced to choose between resolution of motor symptoms without improvement in speech, or preserving speech intelligibility without relief from motor symptoms: “Ceasing stimulation may reverse these exacerbations (of dysarthria), which may be accepted as a therapeutic compromise.” (page 1513).

Looking at laterality of stimulation in recent work, Wang [22, 23] has noted that stimulation of the left STN has worse effects on speech than stimulation of the right STN. All of the patients in her work were unilaterally implanted and right handed, and she used a variety of acoustical and perceptual analysis methods. 

Törnqvist et al. [24] examined the effects of varying different DBS parameters on speech. Comparing each patient's optimal settings to no stimulation, five of 10 patients' speech was the same, while that of four more was worse. Increasing the frequency of electrical stimulation resulted generally in a nonsignificant decrease in number of words judged correct. Increasing stimulation amplitude also reduced the number of words judged correct, while decreasing the amplitude made speech slightly better. Changing which electrode was stimulated created no significant changes. The researchers did not vary pulse width in their study because of its propensity to cause unpleasant side effects. More generally, Montgomery [25] reports that low-frequency DBS of the STN (on the order of 10 Hz) improves speech, while frequencies closer to 100 Hz cause it to deteriorate (while improving general motor function).

Overall, this represents only the beginning of understanding of the changes in speech performance following DBS surgery and therapy, especially with respect to programming strategies and possible tradeoffs between motor improvements and dysarthric side effects. Given that the search for DBS parameters that improve speech is so often subordinate to the search for parameters that improve general motor function, it is vital for the clinician to understand the range of effects on speech he or she can expect from a patient's “optimal” settings.

### 1.2. Goal and Context of the Current Study

This paper reports on the results of acoustic analyses on the speech of patients with PD, both on and off DBS stimulation. The goal of the present study was to gain information on how clinically motivated alterations in DBS settings, made by neurologists whose sole goal is maximizing the motor performance of patients with PD, change speech. This study is part of an ongoing longer-term project to develop a device to measure the speech performance of PD patients on DBS or other therapies. Such a device would allow clinicians to assess, quickly and objectively, whether a provisional set of DBS parameters improves or impairs a patient's ability to communicate, or whether any treatment is having a positive effect on speech. Though certain DBS parameters have more of a positive effect on speech than others, to be useful, such a device must be capable of evaluating speech at the full range of provisional DBS settings a clinician might consider. 

Because the aim of the study was to provide realistic clinical information for practitioners that could potentially apply to any patient with a DBS implant, and because data collection of the speech of patients with DBS was carried out in a clinical setting in the context of patient visits with a strictly therapeutic purpose (to maximize motor benefit, rather than to optimize speech quality or with the experimental needs of this study in mind), several aspects of the study's methodology are unusual.

- Recordings were not made in a sound-treated room but in the room where each patient had his or her DBS implants adjusted. The recording environment used in this study is typical of the recording environment in which the contemplated assessment device would be used.
- A variety of general-purpose microphones was used, similar in frequency response and directivity to the microphones that would be used in the final product.
- Patient-specific factors such as electrode insertion site, time since surgery, or specific DBS settings were under the control of the surgeon and neurologist, and decisions were made based on patient-care considerations rather than the needs of this study. Recent work [26] suggests no association between, for example, speech response and time since diagnosis.
- The number of data points available for a given patient, as well as other factors such as the times of day of the recordings, was determined by the patient's needs and neurologist's treatment decisions.
- The DBS parameter settings represented in the dataset were outside of our control. They were chosen by the neurologist in an attempt to maximize clinical (nonspeech) benefit to the patient. As such, each on-stim session represents a provisional setting at which our anticipated device might be asked to evaluate speech.

Further work is needed to elucidate the effect on different aspects of speech of all of the factors mentioned. In essence, this study was meant to replicate the clinician's situation as much as possible in that he or she must evaluate the speech of every patient whose DBS implants need adjusting, regardless of individual parameter settings, DBS model, or other patient-specific factors. As a companion study and followup to this initial acoustical project, we are undertaking a perceptual study of to determine how well our acoustic measures correlate with intelligibility and how large a change in each measure might be required in order to produce a perceptually noticeable effect.

## 2. Methods

### 2.1. Subjects

Twelve normal-speaking subjects (five female, seven male; age range 26–67) were recorded as controls, once each. 10 patients with PD (two female, eight male; ages 48 to 70) participated in the study. All patients were determined to be good candidates for DBS therapy by a movement disorder neurologist. Inclusion criteria were that all patients presented with idiopathic PD, displayed no other neurological or psychiatric conditions, were at Hoehn and Yahr Stage 3 or 4, received scores of at least 2 on UPDRS items 5 or 18, and had reached a point in their disease where medications caused large and uncomfortable mobility fluctuations. All patients gave written consent to surgical implantation of bilateral electrodes into the STN as well as to participation in the study. It should be noted that at the hospital where the recordings were made, the goal of treatment was to minimize the amount of medication that patients require after surgery; medication was only reinstated after the patient's DBS settings had been optimized if still necessary. In this paper, we report only on the results obtained from patients who discontinued medication therapy after surgery and were treated with DBS only.

### 2.2. Recording Sessions

Subjects were recorded presurgery, both off medication and on medication. Patients were also recorded after surgery, both off DBS stimulation and on stimulation. Some patients were recorded multiple times over periods varying from several weeks to a few months when they came to the clinic for DBS adjustment visits.

Because no on-meds sessions were analyzed here, the treatment states discussed below are “on-stim” (postimplant patient receiving stimulation), “off-stim” (postimplant patient not receiving stimulation), and “normal” (control speakers without PD). Specifically, we compare the off-meds/off-stim sessions with the off-meds/on-stim sessions for each patient. The term “session” is used in this paper to refer to a patient's speech performance at a specific set of DBS settings. That is, if a patient is recorded off-meds and off-stim, then recorded again off-meds and on-stim at a particular set of parameters, and finally again off-meds and on-stim at a different set of parameters, this is considered to be three sessions.

### 2.3. Equipment

Subjects' speech was recorded using head-mounted or desktop microphones and the Marantz PMD-660, a solid-state digital recording device. Recordings (44 kHz, 16 bit, mono) took place in an examination room that was quiet but not sound-treated.

### 2.4. Speech Material Recorded

Two speech tasks were recorded for this study: the bilabial and velar alternating motion rate (AMR) or “rapid repeating” tasks. Participants were instructed to say the syllable “pa” (or “ka”) as quickly and accurately as possible on one breath, like this: “papapapa....”. Both control subjects and patients were given the same instructions. The AMR task specifically assesses the rate, rhythm, and precision of movement of the jaw, lips, and tongue. It also assesses the speaker's ability to coordinate vocal tract movements with laryngeal movements [2, page 7; 28]. 

Three bilabial AMR utterances were produced by each participant during each session and recorded for later repeatability analysis. Because studies by Logemann and Fisher [27], Weismer [28], and Wang et al. [23], indicate that the consonant /k/ poses the most problems for Parkinson's patients, subjects were also asked to perform the velar AMR task once per session, and that utterance was recorded.

### 2.5. Patient Recording Procedure

#### 2.5.1. Presurgical Evaluation Visit

Patient enrollment occurred at the initial presurgical evaluation visit. After entry into the study and prior to surgery, patients participated in two recording sessions. An off-meds session was recorded after discontinuing medication for at least 12 hours, and an on-meds session was recorded 30 to 60 minutes following administration of a typical dose of Sinemet. Sinemet is the Merck brand name for a combination of carbidopa and levodopa. The elimination half-life of levodopa in the presence of carbidopa is about 1.5 hours. The apparent half-life of levodopa taken in the form of Sinemet CR may be prolonged because of continuous absorption [29, 30]. Regardless of an individual patient's specific dose, however, 12 hours is considered enough time for complete washout.

#### 2.5.2. Surgery

The surgical procedure involved bilateral implantation of stimulating electrodes into the STN. Preoperatively, stereotaxic MRI was used to determine the location of the STN in each individual and the best trajectories for the electrodes. Intraoperatively, microelectrode recordings were used to verify and localize STN neurons. Final placement of stimulating electrodes was determined following awake test stimulation for clinical effect and side-effect monitoring, and postoperative MRI and CAT scans were used to verify correct lead placements. Pulse generators and lead extensions were then implanted under general anesthesia.

#### 2.5.3. Postsurgical Visits

Patients returned to the hospital between one and three weeks after implantation. During this visit, they underwent a standard postoperative evaluation regarding common medical issues relating to recovery from anesthesia, incisional healing, and dressing care. Patients had stopped their medical therapy at least 12 hours prior to this visit and were thus in the off-meds state for this visit. 

The patient's DBS device was then turned on and evaluated for clinical effect. Each site of the stimulating electrode was tested individually and voltage was increased to determine threshold boundaries for side effects. A combination of sites were then activated within a voltage range that was below the side-effect threshold and at a level that also produced clinical benefit. An initial session of on-stim utterances was recorded after five-to-ten minutes of acclimation to the initial stimulation.

#### 2.5.4. Adjustment Visit(s)

Patients returned to the clinic as frequently as necessary for adjustments in stimulation to improve clinical benefit. This often required multiple visits during the first several months. For each visit, patients arrived on-stim, at the settings that had been programmed for them at the last visit. Another session of utterances was recorded in this condition.

Stimulation parameters of a patient's implant were then adjusted to maximize clinical benefit. A combination of scores on UPDRS items 23–26, clinician observation, and patient report was used to assess tremor, rigidity, and bradykinesia as measured in limb movements. Speech quality was not assessed or used to adjust the implant settings. 

After each adjustment, the neurologist left the clinic room, while a research assistant, blind to the patient's DBS parameter settings, recorded another complete set of on-stim utterances. The DBS settings corresponding to this new session were noted in the patient's chart.

### 2.6. Audio Data Processing

Recorded utterances were downloaded from the recorder to a USB disk, deidentified to protect participant identity, and brought to a separate facility to be analyzed. Though blinding is generally not necessary for acoustic analyses, researchers at this facility were kept blind to the treatment status and identity of each patient whose speech files were analyzed. Once downsampled and transferred to hard disk, a trained phonetician examined the spectrograms of each utterance to assure data quality and to discard unusable utterances. Reasons for discarding recorded utterances included the presence of electronic or acoustic artifacts or unusual behavior by patients (such as singing or crying). Less than 10% of the total recorded utterances were discarded.

As an ongoing monitoring procedure of the recording conditions and the microphones used, spectrograms of the utterances were examined for any frequency bands between 75 Hz and 8 kHz with markedly lower energies, and none were observed. Further, of all the measures discussed in this paper, only the pitch estimation algorithm (used to identify voiced regions) compares signal energy across frequency bands. Thus, the analyses are otherwise insensitive to details of the spectral profile of microphone response and are generalizable across many different commercially available microphones.

All validated utterances were analyzed automatically by Matlab programs written specifically for each analysis (described below). The same acoustic measurements were applied to all utterances, including those from normal speaking subjects and PD patients. As an initial processing step, recordings were high-pass filtered at 75 Hz, low-pass filtered at 8 kHz, and downsampled to 16 kHz. Though there is useful information in speech at frequencies up to 20 kHz [31], the traditional sampling rate of 16 kHz provides more than enough information for the features analyzed here. Four of the measures used are not frequency-domain measures; the other two concern syllable rate, which is determined using information below 8 kHz. More details concerning the acoustic analysis methods can be found in [32, 33].

## 3. Signal and Statistical Analysis

The velar AMR utterance from each session was analyzed for the following measures: syllable rate, syllable length variability, syllable length patterning, vowel fraction, voice-onset time variability, and spirantization. The three bilabial AMR recordings from each session were analyzed for syllable rate and syllable length variability. Each measure presented below addresses an acoustic characteristic of hypokinetic dysarthria discussed in the literature. Also, the mean value of each measure for the patients with PD off-stim differs from the mean value of the same measure for normal speakers by more than two standard deviations of the normal figures. Each measure is described in more detail below. 

### 3.1. Syllable Rate and Syllable-Length Variability

Consistent speech is important for communication. Variable rate and rapid speech detected in AMRs are both “prominent and/or distinguishing” characteristics of hypokinetic dysarthria [12, page 418]. This observation notwithstanding, Blanchet and Snyder [34] report that of nine studies of AMR rate in PD patients, eight of them found either normal or slow rate for at least some syllable types. Only one, [35], found a “somewhat rapid repetition rate” of monosyllables. 

Speech pathologists typically measure rate by hand as the number of syllables per unit time [35]. In this study, rate was measured automatically by a computer algorithm designed specifically for the purpose. The number of syllables per unit time for the AMR tasks was calculated for each patient utterance and compared to the results for normal subjects as well as to other results for the same patient.

The algorithm identifies AMR syllables by finding the peak energy in the vocalic segment. On either side of this peak are located the acoustic events indicating the beginning and end of glottal vibration; these delineate the beginning and end of the voiced segment. The acoustic events indicating consonant release appear before the beginning of the vowel and are considered the syllable's onset. The whole syllable, therefore, is delineated by the initial event of the consonant release and the final event of cessation of glottal vibration. Syllable rate is defined as the total number of syllables detected, minus one-half syllable, divided by the time between the start of the first syllable and end of the last. Subtracting one-half syllable approximately compensates for the lack of an intersyllable gap after the last syllable. More details on the syllable-detection algorithm can be found in [33].

To assess variability, the relative deviation of syllable length was used instead of standard deviation. This statistic, which is calculated by dividing the standard deviation by the mean, normalizes for differences in syllable length between speakers with and without PD.

### 3.2. Syllable Length Patterning

During a normal AMR, the syllables will not have precisely the same length, but the overall result may sound regular, because the amount of variation will be small; that is, the syllable length will be fairly stable. A histogram of a normal AMR task will show one large peak at the mean syllable length with some spread to either side. Occasionally, PD patients (and some control speakers) use an alternating stress pattern in their AMR tasks. In this case, the histogram will show two peaks with some spread to either side, one at the mean length of the stressed syllables and one at the mean length of the unstressed syllables. When AMR rate is truly irregular, however, no significant peaks will be evident in the histogram of syllable lengths. The standard or relative deviation is inadequate to distinguish between cases where there is an audible pattern in syllable lengths and those where there is no pattern: it will simply show a large value in both cases. 

To discover whether there might be an underlying but inaudible variability in the AMR tasks of PD patients, we used a measure that would be sensitive to syllable length patterns if they existed. This measure, called *negentropy* [36], indicates the degree of clustering in AMR syllable lengths; that is, the number of histogram peaks, measured in bits. Negentropy is high when there is one main syllable length (as in normal AMRs) and when there is a pattern to the syllable lengths (as in the alternating-stress case). It is low when syllable lengths vary randomly and create no pattern to the distribution of lengths.

### 3.3. Vowel Fraction

Once the vowel of each syllable has been located and its onset determined, it is possible to measure the length of time that the vowel lasted as a fraction of total syllable length. In normal speech, as the rate changes, the length of the vowel changes more than the length of the consonant. Thus, if overall speech rate decreases, it is mainly due to lengthening of the vowel; if speech rate increases, it is mainly due to shortening of the vowel. The vowel ratio is a measure designed to assess whether, if the speech of subjects with PD has a different rate than that of control subjects, the syllables change “shape” in the same way that normal speech does.

### 3.4. Voice-Onset Time Variability

Voice-onset time (VOT) is the interval between the release of a stop consonant and the beginning of voicing for the following vowel. Given the prevalence of rigidity and bradykinesia in PD, changes in VOT relative to normal may reflect these symptoms. To calculate VOT, the time between the landmark indicating consonant release and the landmark indicating phonation onset was measured for each syllable in the AMR task. The standard deviation across all syllables was then found.

### 3.5. Stop Consonant Spirantization

During normal speech, consonants are produced by closure of the oral cavity. This closure stops phonation and leads to the silent interval found in stops. When the closure is incomplete, however, air escapes during what should be a silent interval and results in spirantization, or weakening, of the stop [32]. For example, a /t/ spirantized by a patient with PD may sound more like [s]. The unvoiced velar stop /k/ was used for this analysis, as research has shown it to be the most commonly spirantized stop in the speech of PD patients [27, 28].

The algorithm that assessed how much spirantization was present in each of the AMR syllables first found the mean signal amplitude between syllables. This was compared to the peak amplitude of the syllable. The result was a spirantization score ranging from a complete stop, score = 0, to no stop, score = 1. Thus, the lower the spirantization score, the less spirantization, and vice versa.

### 3.6. Statistical Analysis

The value of each measure described above was determined for each patient and for every session, including both on-stim and off-stim speech. Using the mean and standard deviation of the normal speakers' scores on each measure as a reference, a patient's score on each measure was converted to a *z*-score. As we will see below, the measures that showed population-level differences were syllable rate, syllable length variability, syllable length patterning, voice-onset time variability, and spirantization (all except vowel fraction). The absolute values of the *z*-scores of those five measures for each recording session were then averaged to create a composite measurement for each session. This composite measurement represents how far away a patient was from normal at any one session. A comparison of composite measurements between sessions shows how far a patient moved toward or away from normal with a change in DBS parameters, allowing easy comparison from one condition to the next. To compare each patient's overall on-stim performance to their overall off-stim performance, the off-stim session with the median average absolute *z*-score was used.

## 4. Results

A final set of 102 sessions was available for analysis, including normal utterances, patient utterances on-stim, and patient utterances off-stim. Each patient had at least three sessions, but five patients account for particularly large numbers of sessions, between eight and 23 each, because they required multiple adjustments. 

### 4.1. Syllable Rate

The first analysis looked for differences in mean syllable rate between treatment conditions; these results are tabulated in Table 1 for the velar AMR. Speakers with PD generally showed significantly slower syllable rates both on-stim and off, compared to normal controls (normal versus off, *P* < .001, normal versus on-stim, *P* ≪ .001). Additionally, DBS resulted in a small decrease in the mean syllable rate when compared to the off condition though this was not significant. 

These results show that patients have significantly slower syllable rates than normals. Though higher-than-normal rate is a “prominent and distinguishing” characteristic of the speech of speakers with Parkinson's disease [12, page 418], the actual rates from our study are consistent with the studies described in Blanchet and Snyder [34]. Of nine studies described by these authors in which syllable rates on AMR tasks were recorded for individuals with Parkinson's, eight report normal or slow rates; only one reports faster-than-normal syllable rates. Other researchers also report “a relative preservation of speech tempo” in Parkinson's disease, along with a reduction in clarity [37, page 1097].

### 4.2. Syllable Length Variability

**Figure 1:**
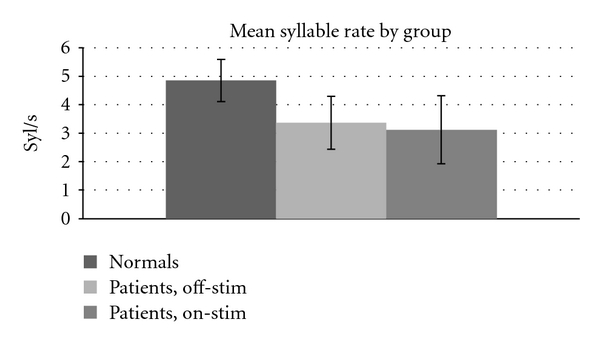
Mean syllable rate across treatment condition. Patients different from normal, *P* ≤ .001.

The second analysis examined differences in syllable length variability within a single utterance for the different groups. This is generally expressed as the standard deviation of syllable lengths within an utterance, as it is in Figure 1. However, since there was a statistically significant difference in syllable length (the reciprocal of syllable rate) between the groups, the relative deviation statistic was chosen for a more specific analysis. The relative deviation normalizes the within-utterance variability to mean within-session syllable length and is more appropriate here. Table 2 displays these values. 

These results indicate that patients have significantly greater variability in length from syllable to syllable, normalized by mean syllable length, than normal speakers (results significant at *P* < .01). Patients on-stim showed slightly greater variability than patients off-stim, but the difference was not significant.

### 4.3. Within-Utterance versus Between-Utterance Variability

Performance of patients with dysarthria can be quite variable, both because of disease processes and because of fatigue associated with compensation for disordered movements. It is, therefore, important to establish how much variability can be expected in the performance of PD subjects between different utterances within a single session. If changes in the mean value of some measure such as syllable rate across multiple repetitions of an utterance within each session are small compared to the variability of the same measure within each utterance, we can conclude that one utterance per recording session will suffice to estimate the performance of a PD patient at a given DBS setting. In terms of the measures we have used so far, this would mean that in order to accept the measures from one utterance as representative of the session, the between-utterance relative deviation in within-utterance mean syllable rate should be small compared to the relative deviation within an utterance. 

To examine this issue, we made recordings of three bilabial AMR utterances from each subject in each session. We measured the duration of each syllable in those utterances then calculated the overall mean syllable rate and relative deviation from each utterance's mean. The first row of Table 3 shows the results for the bilabial AMR task (“papapa”), averaged over all utterances of all sessions of a given treatment group. 

We then calculated the relative deviation in mean rate across utterances within each session, by calculating the standard deviation in mean utterance rate across all utterances in the session, and dividing the result by the mean of the three utterance means. The results, averaged across all sessions of each treatment group, are shown in the second row of Table 3.

Since the between-utterance mean rates (over all three utterances in a session) change much less than the syllable rates within each utterance, we can infer that a patient's performance at a given DBS setting is well estimated by analyzing utterances resulting from single exemplars of appropriate speech tasks.

### 4.4. Syllable Length Patterning

The fourth analysis looked more deeply at the length of syllables in the velar AMR, as produced by the three groups. The question was whether there were underlying but inaudible regularities in the AMR tasks of speakers with PD. Given that the relative deviation of normal speakers' AMR tasks was low, it was not expected that normal speakers would reveal any hidden patterning. However, a large relative deviation can result from two cases: when lengths of syllables are randomly distributed (no patterning) and when lengths of syllables form a pattern. Thus, the syllable length patterning analysis was performed to distinguish between these two cases. Table 4 shows the results of this analysis. 

**Figure 2:**
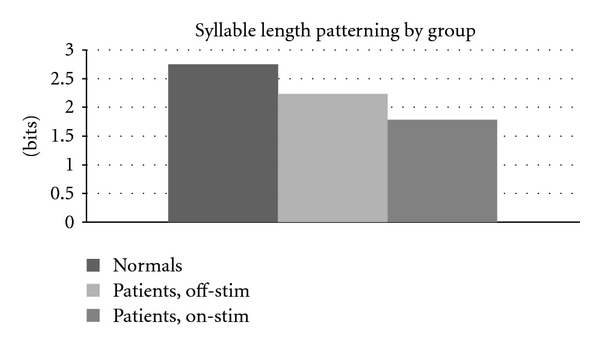
Syllable length patterning, across groups. Lower values indicate random variations in length. Normal values significantly different from patient values (*P* ≤ .002). Patients on-stim significantly different from patients off-stim (*P* < .005).

Analysis of syllable length patterning revealed a significant difference between groups. Differences in patterning were detected between normals and both groups of patients: normals versus patients off-stim (*P* < .002) and normals versus patients on-stim (*P* ≪ .001). Significant differences were also found between patients on and off-stim (*P* < .005). The data for syllable length patterning across treatment states is presented in Figure 2. 

### 4.5. Vowel Fraction

The next measure investigated was the fraction of the syllable duration taken up by the vowel. As seen above, patients with PD have a significantly slower rate than normal. When normal speakers slow their speech, the vowel fraction increases, because the vowel is the part of a CV syllable that lengthens most; consonant length stays nearly the same.  Table 5 shows the vowel fraction for each group of speakers. 

**Figure 3:**
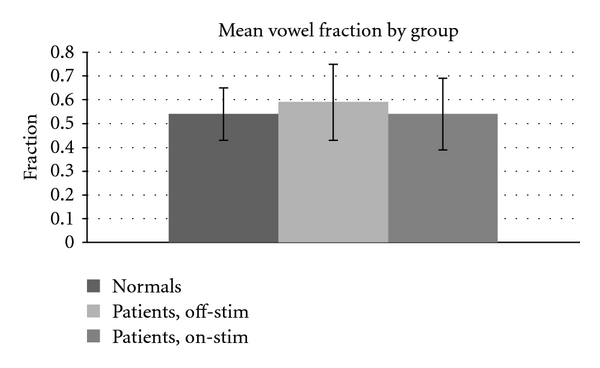
Mean vowel fraction across treatment condition. No comparisons significant.

The Kolmogorov-Smirnov (K-S) test was used instead of the two-tailed *t*-test here, because it does not assume a Gaussian distribution to the underlying variable. Detecting no significant difference indicates a broad similarity of the distributions, not merely an agreement in a particular parameter such as the mean. The K-S test produces *P* > .5 for every pairwise comparison of the states, suggesting that no state differs significantly from any other on this measure. This result is counter to expectations given the slower syllable rate for patients with PD. If their syllables act like those of normal speakers, then their slower rate should result in a larger vowel fraction, since the vowel would lengthen more than the consonant. The fact that this does not happen suggests that the speech of people with Parkinson's disease is not simply a slower version of normal. Instead, it seems that the consonant has lengthened along with the vowel to produce the lower number of syllables per second. Figure 3 shows this result in graphical form. 

### 4.6. VOT Variability

**Figure 4:**
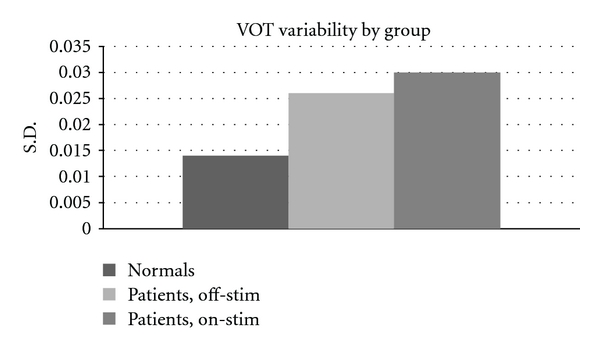
Voice-onset time variability across treatment condition. Normal different from patients, *P* ≤ .01.

Given that the consonants of patients with PD are lengthened in relation to those of normal speakers, deeper investigation into their properties was warranted. To begin with, we examined the consistency or variability with which the speakers produced the voice-onset time of each unvoiced consonant in the velar AMR task. The standard deviation of the VOTs of all the syllables in the task are tabulated in Table 6 and graphed in Figure 4. For both treatments states, patients showed greater VOT variability than normal (*P* < .001 normal versus on-stim; *P* < .01 normal versus off-stim).

The results show that patients, whether on-stim or off, have significantly more variable voice-onset times than do normal speakers.

### 4.7. Spirantization

A final investigation of consonant production in patients with PD focused on the presence and amount of spirantization. Spirantization represents the passage of air through an oral constriction during a time when that constriction should be a complete closure that allows no airflow. The presence of spirantization creates a fricated-sounding stop consonant. 

**Figure 5:**
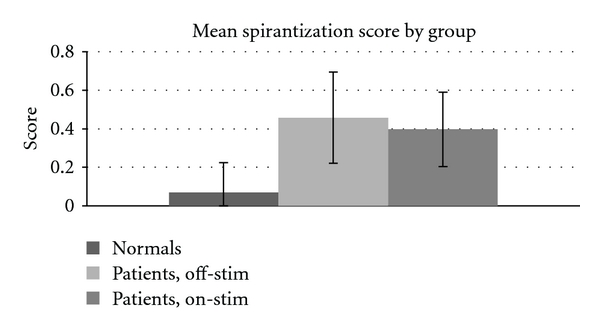
Spirantization score across treatment conditions.

A comparison of spirantization scores revealed significant differences between each patient group and the controls (*P* ≪ .001), as shown in Table 7. No differences were detected between the patient groups. The results of the spirantization measure are also graphed in Figure 5. They suggest that the speech of patients with PD is more spirantized than normal controls and that spirantization is not affected by stimulation. 

### 4.8. Individual Speech Scores

**Figure 6:**
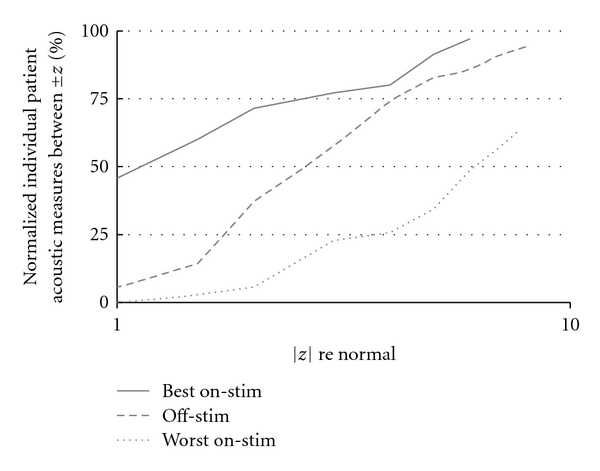
Effect of DBS on acoustic measures of speech: cumulative distribution of individual patient acoustic measures, normalized by scores of control subjects. For ease of visualization, the 0% and 100% extrema of the distributions are not shown.

Using the mean and standard deviation of the normal speakers' scores on each acoustic measure as a reference, each patient's score on the same measure in each session was converted to a z-score. For the 7 patients who had multiple on-stim sessions, and for each acoustic measure other than vowel fraction (which did not vary with treatment), we identified that patient's best on-stim score (smallest |*z*| value), worse on-stim score (largest |*z*| value), and average median off-stim score. Note that a patient's best or worst score for one acoustic measure might well occur at a different DBS setting than the corresponding score for another acoustic measure. Figure 6 shows the cumulative distribution of the three types of scores across all 7 patients and all 5 measures. The three distributions are significantly different, as determined by the K-S test (*P* ≪ .002). 

As shown in Figure 6, almost 50% of the best acoustic scores for patients on-stim are well within the normal range (|*z*| ≤ 1.0). Less than 10% of patients' off-stim scores are in the same range. Thus, stimulation settings were found that did significantly improve most of the identified acoustic measures for most patients (32 out of 35 best on-stim scores were better than the corresponding off-stim score). On the other hand, DBS settings were also evaluated that resulted in significantly degraded acoustic scores: more than half of the worst on-stim scores were greater than six sigma away from normal (|*z*| > 6.0), while only 14% of off-stim scores and just 3% of best on-stim scores were in that extreme range. Only 4 of the 35 worst on-stim scores were better than the patient's corresponding off-stim score. 

### 4.9. Composite Speech Scores

**Figure 7:**
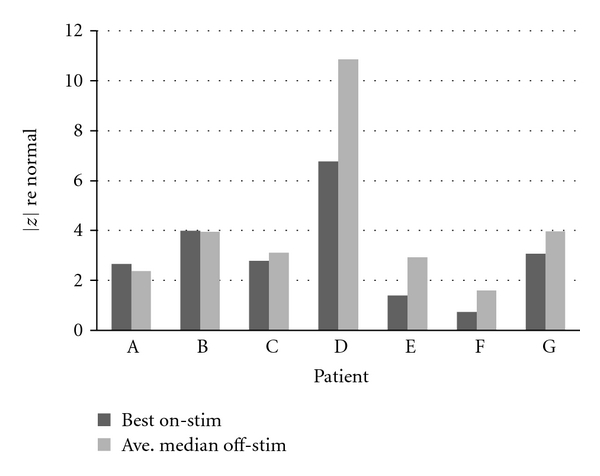
Effect of DBS on speech: best settings.

For the 7 patients with multiple on-stim sessions, their best improvement seen in speech compared to that patient's median off-stim score is detailed in Table 8 and shown in Figure 7. A small |*z*|-score difference represents a DBS setting that had little to no effect on speech. A negative |*z*|-score difference means that a patient's best on-stim settings impaired speech across the five measures compared to when the patient was off-stim. Finally, a positive |*z*|-score difference indicates that a DBS setting was found that improved speech relative to off-stim though, of course, the patient's speech might still be markedly impaired. 

The table and graph show the wide variation between not only in the number of optimization visits a patient experienced, but also in the effect on speech of the parameters tested at each visit. For example, Patient A came back to the clinic only twice after implantation, finding relief from motor symptoms after the second attempt. However, this patient's speech actually deteriorated slightly at those settings, compared to off-stim. By contrast, Patient G came back to the clinic 19 times before an acceptable set of parameters to relieve motor symptoms was found. For this patient, the best effect on speech of all settings attempted was an improvement of approximately one standard deviation, bringing his or her speech at best to 3 standard deviations away from normal. Patient D showed the greatest amount of improvement from off-stim to a session on-stim, over 4 standard deviations' worth, but this patient's speech was also the farthest from normal off-stim, so there was a lot of room for improvement. The last two columns of Table 8 are shown in Figure 7. 

It is also worthwhile to look at the reverse effect of DBS on speech. In the search for optimal motor DBS settings, what was the greatest *deterioration* seen in speech compared to a patient's median off-stim score? Table 9 shows the median off-stim session and the worst on-stim session for each patient with multiple on-stim sessions as well as the difference between that patient's best and worst on-stim sessions. In this table, a negative |*z*|-score difference (compared with off-stim) indicates that the least helpful DBS setting evaluated actually improved speech compared to off-stim.

**Figure 8:**
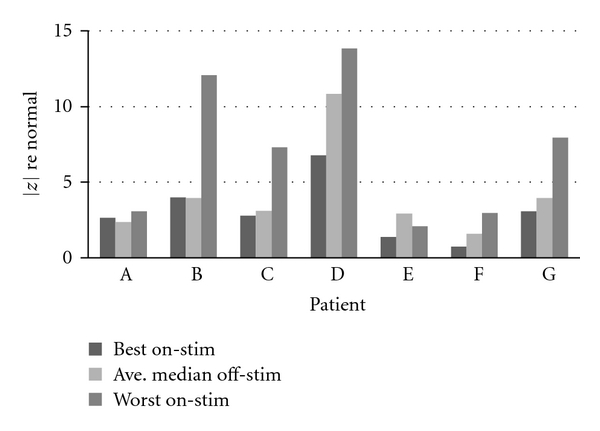
Effect of DBS on speech: best and worst settings.

Figure 8 shows that all patients, except for Patient E, experienced some settings that deteriorated speech relative to off-stim. For Patient E, since all on-stim sessions were better (closer to normal) than off-stim, both differences (best minus off, worst minus off) are negative. Even at this patient's worst settings, his or her speech still benefited from DBS. That is certainly not the case for all the patients, however. Patients B and G, in particular, experienced some settings that made their speech appreciably worse (approximately eight and approximately four standard deviations worse, resp.) than it was off-stim.

## 5. Discussion

The results show that there are acoustic measures which, individually, reveal significant differences between normal speech and Parkinsonian speech. Specifically, syllable length, syllable relative deviation, syllable length patterning, voice-onset time variability, and spirantization differentiate the speech of Parkinson's patients from normal. In addition, syllable length patterning differentiates the speech of PD patients on-stim from off-stim: when on-stim, PD patients' speech shows more random length variations than off-stim. Vowel fraction was the sole measure that showed no significant difference between normal and PD patients' speech, yet this result, too, shows that PD speech is not like normal speech. Whereas in normal speech the vowel lengthens more than the consonant as rate slows, in PD speech the consonant lengthens as well as the vowel. When the five measures that differentiate PD speech from normal are taken together, they show how patients move closer to or farther away from the normal range of function as DBS settings that are intended to relieve motor symptoms change.

These results are important clinically, because, after the limitations in the ability to perform activities of daily living, impairments in communication are often reported as the most important factor reducing quality of life in PD patients (C. Van Horne, personal communication; [12, page 194]). Furthermore, recent work has begun to demonstrate that the DBS settings that best reduce the general motor symptoms of PD are not the same as those that improve speech [26]. In the clinic, neurologists have traditionally used UPDRS item 18 to assess the speech of PD patients during DBS adjustment sessions, but that measure shows poor sensitivity in detecting changes in speech [26, 38]. Thus, there is a need for noninvasive, objective methods for detecting positive or negative changes in speech as a result of DBS therapy. 

The results of the present study are consistent with the results of previous work showing increased variance in syllable length in PD patients receiving DBS stimulation [38] as well as with work demonstrating both positive and negative changes in speech as a result of stimulation [1, 3, 4, 10, 13–24]. These results extend previous work in that they show that within the space of settings that relieve motor symptoms of PD, the potential for degrading speech is greater than the potential for improving it. This cautionary result is supported by other research demonstrating that stimulation of the left hemisphere can have worse effects on speech than stimulation of the right hemisphere [22, 23, 26] and that higher voltage in the left hemisphere is associated with worse speech over the first year after implantation [26]. The present results are also consistent with work showing that stimulation frequency can improve or degrade speech, depending on the frequency range of the stimulation waveform [39]. 

Our results shows that DBS settings chosen for the relief of general motor symptoms of PD can improve speech for most patients, bringing it up to four standard deviations closer to normal for the patients in this study. 75% of all individual acoustic scores could be moved within the normal range, that is, mean ± 2 SD, at *some* DBS setting. For most patients, this was not possible for all measures at the same DBS setting.

The work here also extends previous research on the features of speech that differ between patients with PD and normally speaking subjects, in that it examines several features simultaneously and combines them into a composite measure. Given that speech is comprised of complex motor and acoustic events, it is likely that only a combination of measures will accurately reflect functional differences between normal and disordered speech. Note that the simple composite score we employed simply treats all five measures equally. It is possible that a weighting of scores that was specific to each patient would better express speech functionality. It might be possible to find a DBS setting for many patients that would optimize such a patient-specific composite.

Considering the task of the clinician whose job is to adjust a patient's DBS parameters to maximize his or her function, our results also show that within the range of parameters that are appropriate for the relief of general motor symptoms like tremor, slowness of movement, and rigidity, settings can indeed be found that improve speech significantly. Significantly, the capacity for DBS to worsen speech in the search for device settings adequate to treat the general motor symptoms of Parkinson's disease seems greater than its capacity to improve speech during such searches and is perhaps not unexpected in the context of research showing that the stimulation-frequency ranges for optimal motor and speech function do not overlap [25]. 

These two findings are important both for older implants where there is only a choice between on- and off-stim, and for the newer generation of implants where more than one set of parameters can be stored. In the former case, it may be possible to choose a setting which represents an acceptable compromise between walking and talking, as it were. In the latter case, separate settings, optimized, respectively, for control of motor symptoms and for speech quality, can be stored and selected at will by the patient. 

It would not be possible to identify either compromise or optimal single-purpose settings without consideration of acoustic measures of speech such as the ones used here, because perceptual differences are not always fine or reliable enough to accurately assess small differences in speech. In adjusting DBS parameters, the clinician is faced with the dilemma of needing to optimize them to serve two important functions, while using an instrument (the UPDRS) that is not sensitive enough to detect when speech may be moving away from or toward normal. It is in this context that the results of the present study are the most important. It suggests that for most patients and measures, one or more DBS settings could be found that move that measure of the patient's speech into the normal range. In our study, the setting the clinician settled on for the best improvement in motor function might not, in fact, improve speech, because the settings that improve speech (according to the measures used here) are not always compatible with the settings that relieve tremor and rigidity, and vice versa. Thus, the use of these acoustic measures is an important tool for clinicians who are searching for settings that represent an appropriate compromise between reduced tremor/rigidity and improved speech. The fact that acoustic measures can be obtained noninvasively, objectively, and quickly makes them useful not only in clinical situations but for further research as well.

As noted earlier, no perceptual analysis was performed in this study. Future work will focus on this important topic. Specifically, it is necessary to establish high correlations between intelligibility scores and the acoustic measures chosen to assess patients' speech. Then, acoustic measures which serve as effective proxies for intelligibility may be used in the future acoustic assessment device, reducing dependence on time-consuming perceptual tests of intelligibility. Furthermore, these measures can be used to evaluate the effect of any treatment for hypokinetic dysarthria, not only DBS. Acoustic measures such as those used here cannot replace the trained ear, nor are they meant to, but they can supplement the clinician's ear by documenting improvement in speech as a result of any type of treatment and justify continuing or discontinuing specific therapies.

## 6. Conclusions

This study employed objective, noninvasive acoustic measurements of speech with greater sensitivity than perceptual analyses, especially UPDRS items 5 and 18, to assess the degree to which different DBS settings improve or degrade PD patients' speech relative to normal. Though the DBS settings in this study were selected on the basis of motor effect, not effect on speech, most patients experienced at least one set of stimulator settings that moved their speech into the range of normal as assessed by at least one of the measures used here, considered in isolation. However, during research guided in this manner, the tendency for DBS to degrade speech is substantial and quite possibly greater than its capacity to improve speech. The use of acoustic analysis methods to monitor the speech of PD patients receiving surgical, medical, or behavioral therapy is thus supported as an important adjunct to perceptual methods.

## Figures and Tables

**Table 1:** Syllable rate (syl/sec.) by group. Mean (and standard deviation) are shown for each group. (* = difference from normal significant at *P* ≤ .001).

Normals	Patients, off-stim*	Patients, on-stim*
4.85 (0.74)	3.37 (0.93)	3.12 (1.20)

**Table 2:** Within-utterance relative syllable length variability by group. The average relative deviation is shown for each group for “kakaka” utterances. (* = difference from normal significant at *P* ≤ .01).

Normals	Patients, off-stim*	Patients, on-stim*
0.24	0.33	0.34

**Table 3:** Bilabial within- and between-utterance syllable rate variability by group. The first row shows within-utterance relative deviation or syllable rate. The second row shows between-utterance, within-session mean syllable rate relative deviation. All results are averaged across all sessions of the indicated participant group.

Variability type	Normals	Patients, off-stim	Patients, on-stim
Within-utterance	0.200	0.251	0.558
Between-utterance	0.066	0.083	0.152

**Table 4:** Syllable length patterning (bits) by group. (* = difference from normal significant at *P* < .002).

Normals	Patients, off-stim*	Patients, on-stim*
2.75	2.23	1.79

**Table 5:** Mean (and S.D.) vowel fraction by group.

Normals	Patients, off-stim*	Patients, on-stim*
0.54 (0.11)	0.59 (0.16)	0.54 (0.15)

**Table 6:** VOT variability (S.D.) by group. (* = difference from normal significant at *P* ≤ .01).

Normals	Patients, off-stim*	Patients, on-stim*
0.014	0.026	0.030

**Table 7:** Spirantization scores by group. The mean (and s.d.) are shown for each group. (* = difference from normal significant at *P* ≤ .001).

Normals	Patients, off-stim*	Patients, on-stim*
0.070 (0.154)	0.457 (0.237)	0.397 (0.193)

**Table 8:** Best effect of DBS on speech.

Patient	No. of on-stim sessions	Ave. median off-stim |*z*|	Best on-stim |*z*|	Off-stim minus best on-stim
A	2	2.37	2.65	−0.28
B	3	3.94	3.99	−0.05
C	5	3.10	2.78	0.32
D	6	10.85	6.76	4.09
E	8	2.92	1.39	1.53
F	13	1.59	0.73	0.86
G	19	3.97	3.06	0.91

**Table 9:** Worst effect of DBS on speech.

Patient	Ave. median off-stim |*z*|	Worst on-stim |*z*|	Worst on-stim minus off-stim	Worst on stim minus best on-stim
A	2.37	3.06	0.69	0.41
B	3.94	12.06	8.12	8.07
C	3.10	7.30	4.2	4.52
D	10.85	13.83	2.98	7.07
E	2.92	2.10	−0.82	0.71
F	1.59	2.98	1.39	2.25
G	3.97	7.95	3.98	4.89
